# Meal-induced inflammation: postprandial insights from the Personalised REsponses to DIetary Composition Trial (PREDICT) study in 1000 participants

**DOI:** 10.1093/ajcn/nqab132

**Published:** 2021-06-08

**Authors:** Mohsen Mazidi, Ana M Valdes, Jose M Ordovas, Wendy L Hall, Joan C Pujol, Jonathan Wolf, George Hadjigeorgiou, Nicola Segata, Naveed Sattar, Robert Koivula, Tim D Spector, Paul W Franks, Sarah E Berry

**Affiliations:** Department of Twin Research, King's College London, London, United Kingdom; Department of Nutritional Sciences, King's College London, London, United Kingdom; School of Medicine, University of Nottingham, Nottingham, United Kingdom; Nottingham National Institute for Health Research Biomedical Research Centre, Nottingham, United Kingdom; Jean Meyer USDA Human Nutrition Research Center on Aging at Tufts University, Boston, MA, USA; Department of Nutritional Sciences, King's College London, London, United Kingdom; Zoe Global Ltd, London, United Kingdom; Zoe Global Ltd, London, United Kingdom; Zoe Global Ltd, London, United Kingdom; Centre for Integrative Biology, University of Trento, Trento, Italy; Institute of Cardiovascular and Medical Sciences, University of Glasgow, Glasgow, United Kingdom; Oxford Centre for Diabetes, Endocrinology and Metabolism, Radcliffe Department of Medicine, University of Oxford, Oxford, United Kingdom; Department of Twin Research, King's College London, London, United Kingdom; Department of Twin Research, King's College London, London, United Kingdom; Department of Clinical Sciences, Lund University, Malmö, Sweden; Department of Nutrition, Harvard TH Chan School of Public Health, Boston, MA, USA; Department of Nutritional Sciences, King's College London, London, United Kingdom

**Keywords:** glycoprotein acetylation, postprandial glycemia, postprandial lipemia, visceral fat mass, inflammation

## Abstract

**Background:**

Meal-induced metabolic changes trigger an acute inflammatory response, contributing to chronic inflammation and associated diseases.

**Objectives:**

We aimed to characterize variability in postprandial inflammatory responses using traditional (IL-6) and novel [glycoprotein acetylation (GlycA)] biomarkers of inflammation and dissect their biological determinants with a focus on postprandial glycemia and lipemia.

**Methods:**

Postprandial (0–6 h) glucose, triglyceride (TG), IL-6, and GlycA responses were measured at multiple intervals after sequential mixed-nutrient meals (0 h and 4 h) in 1002 healthy adults aged 18–65 y from the PREDICT (Personalised REsponses to DIetary Composition Trial) 1 study, a single-arm dietary intervention study. Measures of habitual diet, blood biochemistry, gut microbiome composition, and visceral fat mass (VFM) were also collected.

**Results:**

The postprandial changes in GlycA and IL-6 concentrations were highly variable between individuals. Participants eliciting an increase in GlycA and IL-6 (60% and 94% of the total participants, respectively) had mean 6-h increases of 11% and 190%, respectively. Peak postprandial TG and glucose concentrations were significantly associated with 6-h GlycA (*r* = 0.83 and *r* = 0.24, respectively; both *P* < 0.001) but not with 6-h IL-6 (both *P* > 0.26). A random forest model revealed the maximum TG concentration was the strongest postprandial TG predictor of postprandial GlycA and structural equation modeling revealed that VFM and fasting TG were most strongly associated with fasting and postprandial GlycA. Network Mendelian randomization demonstrated a causal link between VFM and fasting GlycA, mediated (28%) by fasting TG. Individuals eliciting enhanced GlycA responses had higher predicted cardiovascular disease risk (using the atherosclerotic disease risk score) than the rest of the cohort.

**Conclusions:**

The variable postprandial increases in GlycA and their associations with TG metabolism highlight the importance of modulating TG in concert with obesity to reduce GlycA and associated low-grade inflammation–related diseases.

This trial was registered at clinicaltrials.gov as NCT03479866.

See corresponding editorial on page 841.

## Introduction

Persistent low-grade inflammation is a common pathogenic feature of many chronic diseases, including cardiovascular disease (CVD), type 2 diabetes (T2D), and other chronic metabolic conditions. Although inflammation can be a consequence of the biological characteristics of an individual, it is chronically (1) and acutely (during the postprandial phase) (2, 3) affected by diet. However, current dietary approaches to lower cardiometabolic risk are not specifically aimed at reducing inflammation. Many of the observed chronic effects of dietary carbohydrates and fats on cardiometabolic disease (4) are underpinned by postprandial excursions in glucose and triglycerides (TGs). These include effects on oxidative stress [generation of reactive oxygen species (ROS)], hemostatic function, lipoprotein remodeling (5, 6), and endotoxemia (7), which trigger an inflammatory response. Although an acute inflammatory response is a physiological defense mechanism, a continually activated response from abnormal postprandial metabolic excursions may result in persistent low-grade inflammation and increased risk of cardiometabolic diseases. Given the significant time spent in the postprandial state (up to ∼18 h/d), attenuating an individual's postprandial inflammatory response may provide a dietary target in cardiometabolic disease prevention.

Several small human studies (*n* = 6–86 participants) have evaluated the effects of food given as single meals (high-fat, high-carbohydrate, or mixed meals) on postprandial inflammatory responses (2, 3). However, these reports are inconsistent, which may be attributable to the inflammatory mediators investigated [typically TNF-α, IL-8, IL-6, and C-reactive protein (CRP)]. IL-6 is the only inflammatory marker that has been shown to consistently change postprandially, which may be a consequence of its tissue synthesis and time scale of initiation compared with other markers (3). It is also unclear whether different features of the postprandial metabolic response (e.g., response duration compared with peak concentration) differentially affect postprandial inflammation. Moreover, the mechanisms linking metabolic responses and postprandial inflammation have not been explored in the context of wider biological determinants.

Until recently, no inflammatory marker that was consistently responsive to food, relatively stable within individuals, and clinically relevant was known. Glycoprotein acetylation (GlycA) is an emerging inflammatory biomarker (8, 9), arising from signals detected by NMR spectroscopy (10, 11) from glycan groups of certain acute-phase glycoproteins (12). Elevated GlycA concentrations are predictive of fatty liver (13), T2D (14), some cancers (12), CVD, and mortality (15, 16). GlycA exhibits low intraindividual variability, is independent of CRP, has low inter- and intra-assay variability (15), and is suggested to better reflect inflammation than traditional inflammatory markers such as CRP and IL-6 (10, 11, 16). However, to date there are no published studies on the postprandial GlycA response.

PREDICT (Personalised REsponses to DIetary Composition Trial) 1 (*n* = 1002) aimed to predict individual variations in postprandial TG and glucose responses to standardized meals in a tightly controlled setting and with multiple postprandial assessments. In this article, we characterize postprandial inflammatory responses using traditional (IL-6) and emerging (GlycA) biomarkers of inflammation and dissect their determinants with a focus on postprandial glycemia and lipemia.

## Methods

### The PREDICT 1 study

**FIGURE 1:**
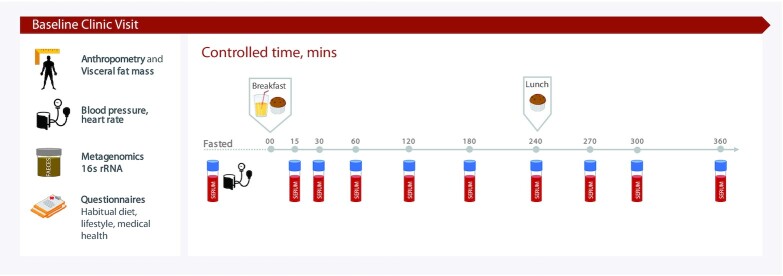
Experimental design. Participants arrived fasted for their clinic visit and were given a standardized breakfast (0 h, metabolic challenge meal, 86 g carbohydrate, 53 g fat) and lunch (4 h, 71 g carbohydrate, 22 g fat). Concentrations of glucose, triglycerides, insulin, IL-6, and glycoprotein acetylation were determined from venous blood collected at multiple time points postprandially. Anthropometric, fasting biochemistry, microbiome, and habitual dietary measurements were also made. rRNA, ribosomal RNA.

The PREDICT 1 clinical trial (NCT03479866) aimed to quantify and predict individual variations in metabolic responses to standardized and free-living meals according to the full protocol, published elsewhere (17). Briefly, the PREDICT 1 study was a single-arm, single-blinded dietary study conducted between June 2018 and May 2019 (**Figure 1**); 1002 generally healthy adults, aged 18–65 y, from the United Kingdom {nontwins, and identical [monozygotic (MZ)] and nonidentical [dizygotic (DZ)] twins} were enrolled into the study (17) and completed baseline clinic measurements [see the Consolidated Standards of Reporting Trials (CONSORT) flow diagram in **Supplemental Figure 1**]. The study consisted of a 1-d clinical visit (day 1) at baseline followed by a 13-d at-home period. Primary outcomes are reported elsewhere (18, 19) and include gut microbiome profile, blood lipids and glucose, sleep, physical activity, and hunger and appetite assessment. Data for the secondary outcomes of inflammation (IL-6 and GlycA) measured at the baseline visit only are reported in this article. At baseline (day 1), participants arrived fasted and were given a standardized metabolic challenge meal for breakfast (0 h; 86 g carbohydrate, 53 g fat, 16 g protein, as a muffin and milkshake) and a test lunch (4 h; 71 g carbohydrate, 22 g fat, 10 g protein, as a muffin). The fat was high-oleic sunflower oil: 85% oleic acid (18:1n–9) and 8% linoleic acid (18:2n–6). Fasting and postprandial (0–6 h) venous blood was collected to determine concentrations of glucose, TG, IL-6, and GlycA (NMR). Stool samples, anthropometry, and a questionnaire for habitual diet were also obtained. Habitual diet information was collected using the European Prospective Investigation into Cancer and Nutrition (EPIC) FFQ (20) to capture average intakes in the past year. The recruitment criteria, outcome variables, and sample collection and analysis procedures relevant to this article are described elsewhere (17). The trial was approved in the United Kingdom by the Research Ethics Committee and Integrated Research Application System (IRAS 236407) and was run in accordance with the Declaration of Helsinki and Good Clinical Practice.

### Exposure measurements

The methods for biochemical and microbiome analyses are described in full elsewhere (17). Briefly, participants were cannulated, and venous blood was collected at fasting (before the test breakfast) and at 9 postprandial time points (15, 30, 60, 120, 180, 240, 270, 300, and 360 min). Plasma glucose and insulin were measured at all time points and serum TG was measured at hourly intervals. Fasting samples were analyzed for lipid profile, thyroid-stimulating hormone, alanine aminotransferase, liver function panel, and full blood count. Assays were performed by Affinity Biomarkers Labs.

Visceral fat mass (VFM) was measured using DXA-based visceral fat measurements. DNA for 16S ribosomal RNA (rRNA) sequencing was isolated by QIAGEN Genomic Services using DNeasy^®^ 96 PowerSoil^®^ Pro. Optical density measurement was done using spectrophotometer quantification (Tecan Infinite 200). For 16S sequencing, the V4 hyper-variable region of the 16S rRNA gene was then amplified at Genomescan. Libraries were sequenced for 300-bp paired-end reads using the Illumina NovaSeq6000 platform. In total, 9.6 Pbp were generated and raw reads were rarefied to 360k reads/sample.

Six features of the glycemic and lipemic postprandial response were analyzed to examine different traits of the postprandial response. Traditionally studies have relied on 0–2 h glucose incremental AUC (iAUC) or 0–6 h TG iAUC when examining glycemic and lipemic associations with disease; however, the importance of the shape of the curve and different features of response for different pathophysiologies is gaining recognition (21). We therefore selected time points to reflect the “total” postprandial response (iAUCs), the peak response (Cmax), the rate of absorption/digestion (Tmax), and the most dynamic part of the curves (for glucose the 30- and 60-min rise from fasting and for TG the 4- and 6-h rise from fasting). The following variables were therefore used in the analysis: for glycemia: 2-h iAUC (Glu_2hiAUC_), 1-h iAUC (Glu_1hiAUC_), fasting (Glu_fasting_), 1-h rise from fasting (Glu_1h-rise_), 30-min rise from fasting (Glu_30min-rise_), and maximum concentration in the first peak at 0–2 h (after breakfast, 1^st^Glu_max_) and second peak at 4–6 h (after lunch, 2^nd^Glu_max_); for lipemia: 6-h iAUC (TG_6hiAUC_), fasting (TG_fasting_), 6-h rise from fasting (TG_6h-rise_), 0–6 h maximum concentration (TG_max_), 4-h concentration (TG_4h_), and 6-h concentration (TG_6h_).

### Outcome measurements

GlycA and IL-6 concentrations were quantified at 3 time points from venous blood at fasting, 4 h postprandial, and 6 h postprandial. GlycA was measured using a high-throughput NMR metabolomics (Nightingale Health) 2016 panel, with a CV of 1.1% (22). Details of the experimentation and epidemiologic applications of the NMR metabolomics platform have been reviewed previously (22, 23). IL-6 was measured by Affinity Biomarkers Lab using a Sandwich Immunoassay by Meso Scale Diagnostics, with an intra-assay CV of 4% within the same run.

### Statistical analysis

Values are expressed as either mean ± SD or median [IQR]. The ln of IL-6 for all 3 time points (+1) was calculated to normalize data distributions. Pearson's *r* was used to determine the relation of different features of the glycemic and lipemic response with both GlycA and IL-6 at all time points (fasting, 4 h, and 6 h). Interindividual variability for each outcome (IL-6 and GlycA) was assessed using Levene's test of variance heterogeneity. TG_max_ and Glu_max_ were used as independent factors in multivariable linear regression models to predict GlycA at 6 h postprandial. Multicollinearity was assessed by evaluating variance inflation factors at each step (considered high when >10) (24). To determine which features of the glycemic and lipemic responses (separately, *n* = 6 for both; combined, *n* = 12) best predicted GlycA (fasting, 4 h, 6 h, 6-h rise), a random forest (RF) model was applied with cross-validation (25). This method fits a large number of classification trees to a data set, then combines the predictions from all trees to present a final predictive model that ranks variables by their predictive power. However, this model does not provide mechanistic insight and may mask variable interaction and nonlinearity. For the evaluation of our models we have used *R*^2^ and *Q*^2^ (an estimate of the predictive ability of the model calculated by cross-validation). A negative *Q*^2^ means the model is not at all predictive.

Receiver operating characteristic (ROC) curves were constructed and the AUC and its 95% CI were calculated to assess the discriminatory power and incremental ability of 1^st^Glu_max_ compared with 2^nd^Glu_max_, 1^st^Glu_max_ compared with TG_max_, and 2^nd^Glu_max_ compared with TG_max_ to detect inflammatory risk (GlycA of 70% was applied as a cutoff). Values of AUC range from 0.5 to 1, with 0.5 indicating no discrimination and 1 indicating perfect discrimination (26). The postprandial increase in inflammatory markers was defined as the 6-h concentration minus the fasting concentration. The 6-h rise was selected owing to the kinetics of the inflammatory response previously reported (3) and the 6-h limit of blood sampling in the current study. The atherosclerotic cardiovascular disease (ASCVD) score was calculated as described previously (27). Repeated-measures ANOVA was used to derive the interaction term between our outcomes (GlycA and IL6) and age and sex. A *P* value < 0.05 was considered statistically significant. Statistical analysis was performed in R Environment for Statistical Computing version 3.5.1 (R Foundation for Statistical Computing; https://www.R-project.org/).

### Mendelian randomization

The **Supplemental Methods** describe the full methods for this section. Briefly, 2-sample Mendelian randomization (MR) was undertaken using summary statistics from published genome-wide association studies (GWASs) to model the causal relations between visceral fat, fasting TG, and fasting glucose (exposures) and fasting GlycA (outcomes) according to published methods (28). The Supplemental Methods report specific information on the genetic instrumental variables [single nucleotide polymorphisms (SNPs)] used here, sensitivity analyses, and other statistical methods.

## Results

### Participant characteristics

A total of 1002 generally healthy adults from the United Kingdom [including nontwins and identical (MZ; *n* = 183 pairs) and nonidentical (DZ; *n* = 47 pairs) twins] completed baseline clinical measurements, dietary assessment, and the sequential test meal challenge [CONSORT diagrams and eligibility criteria are described elsewhere (16)]. **Table 1** summarizes descriptive characteristics of the study participants. Participants were aged between 18.5 and 65.9 y (mean: 45.5 ± 11.8 y) with a mean BMI of 25.6 ± 5.0 kg/m^2^.

**TABLE 1:** Descriptive characteristics of study participants^1^

Variable	Mean ± SD	Median [IQR]
Sex, *n* (M/F)	279/723	
Age, y	45.6 ± 11.9	46.98 [37.57–54.34]
Weight, kg	72.88 ± 15.27	70.28 [61.90–81.30]
Height, cm	168.53 ± 10.51	167.60 [162.30–174.00]
BMI, kg/m^2^	25.59 ± 5.02	24.58 [22.29–27.87]
Waist circumference, cm	85.86 ± 12.93	85.00 [76.00–93.50]
Hip circumference, cm	101.60 ± 10.54	100.00 [95.00–107.00]
Waist-to-hip ratio	0.84 ± 0.08	0.84 [0.79–0.90]
Diastolic pulse, mm Hg	75.78 ± 10.14	75.50 [69.00–82.00]
Systolic pulse, mm Hg	124.26 ± 14.58	123.00 [114.50–132.75]
Alanine aminotransferase, U/L	23.24 ± 11.57	20.40 [16.40–26.08]
Total cholesterol, mmol/L	4.94 ± 0.98	4.87 [4.29–5.55]
HDL cholesterol, mmol/L	1.62 ± 0.41	1.59 [1.32–1.87]
LDL cholesterol, mmol/L	3.07 ± 0.96	2.97 [2.35–3.67]
Non-HDL cholesterol, mmol/L	3.28 ± 1.00	3.17 [2.52–3.90]
Total cholesterol:HDL cholesterol ratio	3.21 ± 0.95	3.06 [2.49–3.69]
Insulin, mU/L	6.13 ± 4.27	5.22 [3.62–7.40]
C-peptide, μg/L	1.19 ± 0.51	1.07 [0.85–1.40]
HbA1c, %	5.47 ± 0.28	5.50 [5.30–5.70]

^1^*n* = 1002. HbA1c, glycated hemoglobin.

### Characterizing the postprandial inflammatory response

**FIGURE 2:**
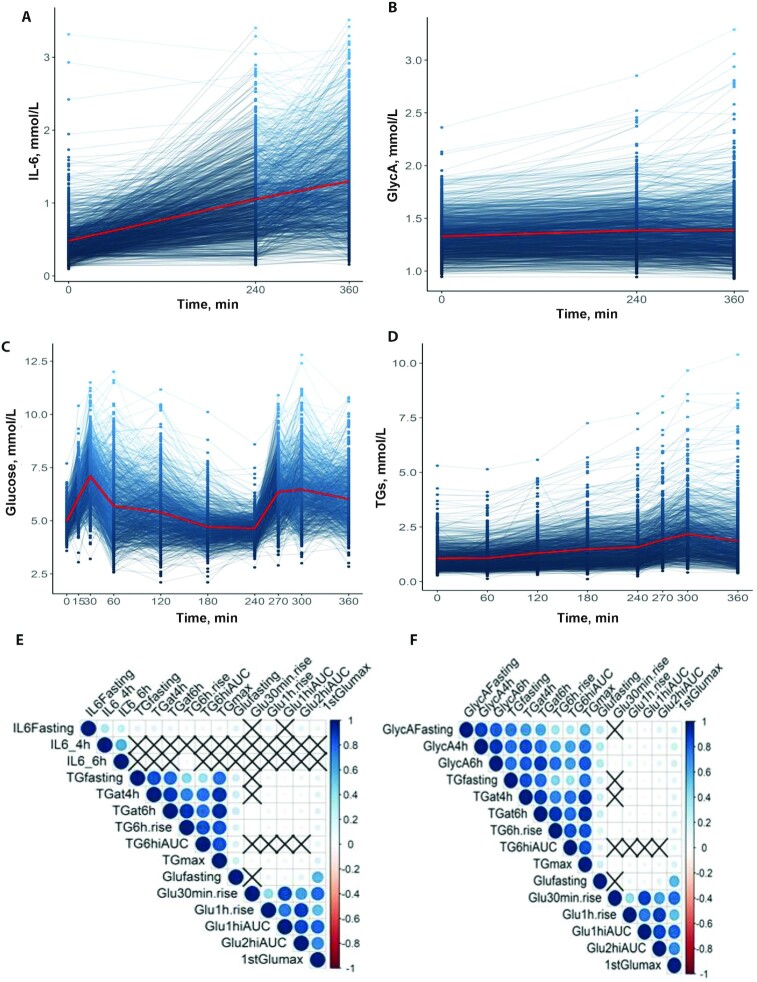
Postprandial changes in IL-6, GlycA, glucose, and TG concentrations (standardized meals consumed at 0 h and 4 h). All *n* = 1002. (A) Fasting, 4 h, and 6 h postprandial concentrations of IL-6 [ln of IL-6 for all 3 time points (+1)]; (B) fasting, 4 h, and 6 h postprandial GlycA concentrations; (C) fasting, 15, 30, 60, 120, 180, 240, 270, 300, and 360 min glucose concentrations; (D) fasting, 60, 120, 180, 240, 270, 300, and 360 min TG concentrations; (E, F) correlation of features of the postprandial glycemic and lipemic responses with fasting and postprandial (4 and 6 h) inflammatory (IL-6 and GlycA) responses (X = nonsignificant correlation). GlycA, glycoprotein acetylation; TG, triglyceride.

IL-6 and GlycA concentrations were measured in the tightly controlled clinic setting at fasting, and after the sequential test meal challenge at 4 h and 6 h postprandial, to assess postprandial changes. IL-6 increased significantly (by 169%) from fasting values (0.48 ± 0.28 mmol/L) and reached peak concentrations at 6 h (1.29 ± 0.61 mmol/L; *P* < 0.001) (**Figure 2**A). In 94% of participants IL-6 increased above fasting concentrations with a mean increase of 0.87 mmol/L (189%) (from 0.46 ± 0.24 to 1.33 ± 0.60 mmol/L). After the first meal (0–4 h), IL-6 concentrations increased for 90.9% and decreased for 8.1% of participants, whereas after the second meal (4–6 h) 71.6% increased and 7.8% decreased from 4-h concentrations. GlycA concentrations increased by a mean of 4.5% (from 1.32 ± 0.18 mmol/L at fasting to 1.38 ± 0.28 mmol/L at 6 h after the meal; *P* < 0.001) (Figure 2B). However, fewer participants elicited a postprandial increase in GlycA than in IL-6; in 60% of participants GlycA increased above fasting concentrations with a mean increase of 10.5% (from 1.34 ± 0.19 mmol/L [IQR: 1.20–1.45 mmol/L] to 1.48 ± 0.30 mmol/L [IQR: 1.28–1.62 mmol/L])—a magnitude which may be clinically relevant for disease risk and mortality (29, 30). For IL-6, women had significantly higher concentrations at fasting and postprandially than men (*P* < 0.001) (**Supplemental Table 1**). However, for GlycA, males had significantly higher concentrations fasting and postprandially (*P* < 0.001). There was no impact of age on IL-6, whereas GlycA concentrations increased with age (*P* < 0.001) (**Supplemental Table 2**). Levene's test of variance heterogeneity revealed that the interindividual patterns of response for each outcome varied greatly for IL-6 (*P* < 0.001) and GlycA (*P* < 0.001) concentrations. GlycA showed greater variation postprandially (6-h CV: 20.2%) than for fasting values (fasting CV: 13.6%), whereas this was not the case for IL-6 (6-h CV: 47.2%; fasting CV: 58.3%), suggesting that postprandial GlycA concentrations may provide better discrimination of an individual's inflammatory tolerance than fasting GlycA concentrations and IL-6 values.

There was a modest correlation between fasting IL-6 and fasting GlycA (*r* = 0.349, *P* < 0.001) and postprandial GlycA at 4 h (*r* = 0.309, *P* < 0.001) and 6 h (*r* = 0.275, *P* < 0.001). However, there was no significant correlation between postprandial IL-6 (4 h and 6 h) and GlycA (fasting or postprandial at 4 h and 6 h; all *P* > 0.326) (**Supplemental Table 3**), highlighting the different patterns of response and potentially divergent food-induced mechanisms of initiation for these 2 inflammatory factors.

Postprandial lipemic and glycemic responses measured during the corresponding postprandial phase [reported previously (18)] were also highly variable between individuals (Levene's test; *P* < 0.001 for all) (Figure 2C, D). Glucose concentrations (Glu_fasting_: 4.91 ± 0.51 mmol/L) peaked (1^st^Glu_max_) at 30 min postprandially after the first meal (30 min: 7.01 ± 1.18 mmol/L, increasing by 42.7%) and again at 5 h (2^nd^Glu_max_), i.e., 1 h after the second meal (6.47 ± 1.41 mmol/L, increasing by 31.7%). TG concentrations (TG_fasting_: 1.05 ± 0.53 mmol/L) peaked at 5 h postprandial (TG_max_: 2.11 ± 1.09 mmol/L, increasing by 100.9%) and remained significantly above fasting values at 6 h (1.85 ± 1.16 mmol/L, *P* < 0.001).

### Relation between features of postprandial lipemia, postprandial glycemia, and inflammatory response

To investigate which traits of the lipemic and glycemic responses (e.g., iAUC or Cmax) were most closely correlated with the postprandial inflammatory response (Figure 2E, F), we selected key postprandial features to reflect different pathophysiological parameters of the postprandial response, as outlined in the Methods. Weak correlations (unadjusted) were observed between fasting IL-6 and fasting TG (*r* = 0.155) and glucose (*r* = 0.160; both *P* < 0.001). However, postprandial IL-6 (4 h and 6 h) was not significantly correlated with any features of the postprandial glucose or TG responses (all *P* > 0.095). Fasting GlycA was correlated strongly with fasting TG (*r* = 0.751) and weakly with glucose (*r* = 0.293), as well as moderately correlated with postprandial TG_at4h_ (*r* = 0.654) and weakly with 1^st^Glu_max_ (*r* = 0.217) measures (all *P* < 0.001). Postprandial GlycA (6 h) was also highly correlated with postprandial TG (TG_max_: *r* = 0.832; TG_at6h_: *r* = 0.816), and weakly correlated with glucose (1^st^Glu_max_: *r* = 0.239; all *P* < 0.001). Further, the 6-h rise in GlycA was strongly correlated with postprandial TG (TG_6h rise_: *r* = 0.884; TG_max_: *r* = 0.748; TG_at6h_: *r* = 0.830) and weakly correlated with glucose (1^st^Glu_max_: *r* = 0.152; all *P* < 0.001).

### Predicting postprandial inflammatory responses after a mixed meal

Owing to its strong association with multiple features of lipemia and glycemia (compared with IL-6), GlycA was used as the inflammatory biomarker for the remaining analyses. Accordingly, we used machine learning to assess which features of the lipemic and glycemic responses influenced the postprandial inflammatory GlycA response. Input features (*n* = 13) were, for glycemia (*n* = 7): Glu_2hiAUC_, Glu_1hiAUC_, Glu_1h rise_, Glu_30min rise_, 1^st^Glu_max_, 2^nd^Glu_max_, and Glu_fasting_; and for lipemia (*n* = 6): TG_6hiAUC_, TG_6h rise_, TG_max_, TG_fasting_, TG_at4h_, and TG_at6h_. Machine learning (RF) revealed that lipemic features were stronger predictors of the postprandial GlycA response than glycemic features, and that GlycA concentration was mainly determined by the corresponding TG feature; e.g., fasting GlycA concentration was mainly determined by the fasting TG concentration (total model: *R*^2^ = 0.56 and *Q*^2^ = 0.54). Moreover, the same was found postprandially: TG_at4h_ for GlycA at 4 h (total model: *R*^2^ = 0.73 and *Q*^2^ = 0.74); TG_at6h_ for GlycA at 6 h (total model: *R*^2^ = 0.80 and *Q*^2^ = 0.80); and TG_6h rise_ for 6-h rise in GlycA (total model: *R*^2^ = 0.72 and *Q*^2^ = 0.76). The performance and generalizability of the machine learning model were higher for postprandial GlycA concentrations than for fasting GlycA, showing that we can predict the highly variable postprandial GlycA concentrations with greater accuracy than fasting values.

### Independent predictors of the postprandial inflammatory response

The postprandial phase is highly dynamic, involving a complex interplay between simultaneous postprandial fluxes in glucose and TG from mixed meals. Therefore, to disentangle the effect of the glucose and TG responses, we evaluated the independent role of lipemia and glycemia [TG_max_, and glucose peaks (1^st^Glu_max_ compared with 2^nd^Glu_max_)] in predicting 6-h GlycA concentration, using multiple regression analysis (**Supplemental Figure 2A–C, Supplemental Table 4**). Owing to the sequential meal study design and overlapping glucose and TG peaks (as occurs in real life), we also dissected the contribution of the first glucose peak (1^st^Glu_max_; between fasting and 2 h), second glucose peak (2^nd^Glu_max_; between 4 h and 6 h), and TG peak (TG_max_; between 0 and 6 h) in independently predicting the 6-h GlycA concentrations. In a model including TG_max_ and 1^st^Glu_max_, both features were significantly and independently associated with 6-h GlycA (*R*^2^ = 0.70). In a second model including only glucose (1^st^Glu_max_ and 2^nd^Glu_max_) (*R*^2^ = 0.057), just the 1^st^Glu_max_ was a significant and independent predictor of 6-h GlycA, suggesting a delayed initiation of GlycA by glucose. In a third model with the coinciding 2^nd^Glu_max_ and TG_max_, both TG and glucose independently and significantly predicted 6-h GlycA (*R*^2^ = 0.69). Finally, ROC-AUC analysis revealed that the TG_max_ was more informative of postprandial 6-h GlycA than both the first and second glucose peaks (Supplemental Figure 2A–C).

### Potential mechanisms underlying postprandial inflammation

**FIGURE 3:**
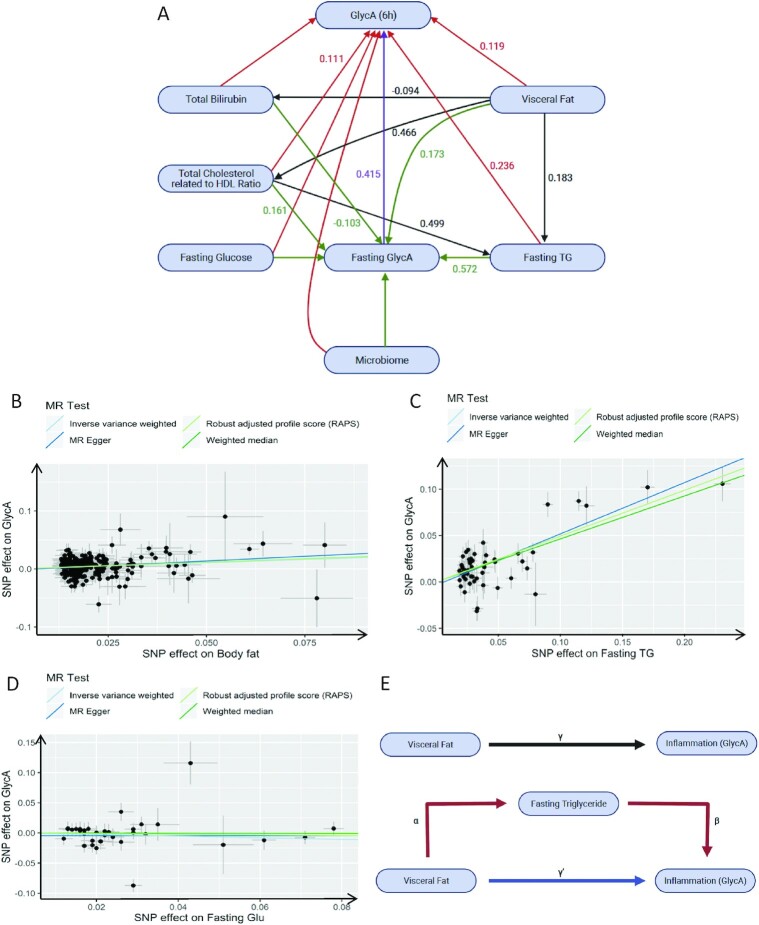
Mechanisms underlying postprandial inflammation. (A) Structural equation model to determine the underlying mechanism of postprandial GlycA (6-h) concentrations. Model definitions, with rectangles representing manifest nodes and arrows indicating regression coefficients pointing toward an outcome of regression (standardized β value mentioned on each arrow only for significant associations); *n* = 1002. (B–D) Scatter plots of the causal effect of (B) visceral fat on fasting GlycA, (C) fasting TG on fasting GlycA, and (D) fasting glucose on fasting GlycA. (E) Network MR. Mediation model for the association between visceral fat and fasting GlycA, with fasting TG as mediator. Path α represents the regression coefficient for the association of visceral fat with fasting TG: “action theory.” Path β represents the regression coefficient for the association of fasting TG with fasting GlycA: “conceptual theory.” The product of regression coefficients α and β (α*β) represents the mediated effect (indirect effect) of fasting TG. Path γ represents the simple total effect of visceral fat on fasting GlycA: “total effect.” GlycA, glycoprotein acetylation; MR, Mendelian randomization; RAPS, robust adjusted profile score; SNP, single nucleotide polymorphism; TG, triglyceride; PCA, principle component analysis.

In addition to the impact of glycemia and lipemia on postprandial inflammation we investigated the relative impact of multiple input variables from our rich data set using a structural equation model (SEM). To decrease data dimensionality [including serum measures, anthropometrics (Table 1), dietary intake (**Supplemental Table 5**), and the top 10 microbiome principle component analysis], we applied machine learning (RF) to select significant predictors of postprandial GlycA (6 h) for inclusion in the SEM. The inclusion of postprandial lipemia and glycemia in the model resulted in a poor-fit model (invalid model) and they were therefore excluded from the final model (**Figure 3**A), to enable us to explore other determinants in a valid model. Our final model demonstrated a good fit [χ^2^: 146.3; comparative fit index (CFI): 0.995; root mean square error of approximation (RMSEA): 0.131; Tucker–Lewis index (TLI): 0.932] and effect estimates are presented in **Supplemental Table 6**. Fasting TG (mmol/L) and VFM had the strongest relation with fasting GlycA (mmol/L) (β = 0.572 and 0.173, respectively) and postprandial GlycA (mmol/L) (6 h) (β = 0.236 and 0.119, respectively) (all *P* < 0.001). Of note, the association between fasting glucose and either fasting GlycA (mmol/L) (β = 0.028, *P* = 0.135) or postprandial GlycA (mmol/L) (6 h) (β = 0.016, *P* = 0.45) was not statistically significant.

### MR

MR analysis was then undertaken to obtain unconfounded estimates of the causal association of genetically determined exposures (VFM, TG, and glucose) and the outcome (GlycA). Fasting GlycA, TG, and glucose were used owing to an absence of postprandial GWAS data; however, our SEM revealed that fasting GlycA was the strongest predictor of postprandial GlycA and we propose that the effect of fasting TG and fasting glucose on postprandial GlycA is mediated in part by fasting GlycA. All instrumental variables (SNPs) had *F* statistics >20 (31). **Supplemental Table 7** presents the results, expressed as the β-coefficients for visceral fat and fasting TG and glucose concentrations per 1-SD increase in fasting GlycA. Individuals with genetically higher visceral fat [inverse‐variance weighted (IVW): β = 0.217, *P* = 6.634e-09; Figure 3B] and fasting TG concentrations (IVW: β = 0.494, *P* = 6.915e-26; Figure 3C) had a significantly greater fasting GlycA, whereas fasting glucose had no significant causal effect on fasting GlycA (IVW: β = −0.132, *P* = 0.294; Figure 3D).

Supplemental Table 7 also shows heterogeneity results and horizontal pleiotropy (where a variant has an effect on other traits outside of the pathway of the GlycA) bias. Estimations based on both MR Egger and IVW for visceral fat were >0.05, indicating a low chance of heterogeneity (IVW: *P* = 0.109; MR Egger: *P* = 0.108). Whereas, there was heterogeneity for the impact of fasting TG (IVW: *P* = 2.958e−05) and fasting glucose (IVW: *P* = 2.382e−09) on fasting GlycA. The results of the Mendelian Randomization Pleiotropy RESidual Sum and Outlier (MR-PRESSO) did not indicate any outliers for all the estimates. The horizontal pleiotropy test, with negligible Egger regression intercept, also indicated a low likelihood of pleiotropy for all of our estimations (all *P* > 0.335). The results of the MR robust adjusted profile score (MR-RAPS) were identical with the IVW estimates, highlighting again a low likelihood of pleiotropy (Supplemental Table 7). The results of the leave-one-out method demonstrated that the links were not driven by single SNPs.

### Network MR

To evaluate the extent to which fasting TG mediates the effect of visceral fat on GlycA variation we performed network MR (Figure 3E). These analyses revealed that the effect of visceral fat on fasting TG variation was statistically significant (IVW: β = 0.127, SE = 0.03, *P* = 0.000024), whereas the effect of fasting TG on visceral fat variation was not (IVW: β = −0.052, SE = 0.02, *P* = 0.057), suggesting a predominantly unidirectional causal relation. The effect of a 1-SD increase in visceral fat on fasting GlycA was 0.22 (standardized-β). The effect of a 1-SD increase in visceral fat on fasting TG was 0.13 (standardized-β) and the effect of a 1-unit increase in fasting TG on fasting GlycA was 0.49 (standardized-β). Thus, the mediated effect of fasting TG was 0.13 × 0.50 = 0.065. The mediated proportion was 0.065/0.22 = 29%, suggesting therefore that ∼29% of the effect of visceral fat on inflammation is mediated by the metabolic processes that underlie fasting TG.

## Discussion

The postprandial inflammatory response may affect the pathophysiology of many chronic diseases. In the largest postprandial inflammation study to date, we *1*) evaluated the association of different features of the food-induced TG and glucose responses with postprandial inflammation, *2*) determined the main predictors of postprandial inflammation, and *3*) explored potential mechanisms underlying postprandial changes in inflammation. Postprandial lipemia was a stronger predictor of postprandial inflammation (measured by GlycA) than was postprandial glycemia. Visceral fat, partly mediated by fasting TG, was a key causal determinant of this postprandial inflammatory response, supporting current evidence that management of obesity and TG concentrations (via lifestyle or drugs) will reduce chronic inflammatory burden, a key factor in the pathogenesis of cardiometabolic diseases.

Several small-scale human studies have evaluated the effects of high-fat (3) or high-carbohydrate (32) meals on inflammatory responses. However, there has been no consensus regarding the timing, magnitude, and mechanism underpinning postprandially stimulated inflammation or the most relevant food-induced inflammatory biomarkers. Further, there is little agreement on the impact of postprandial glycemia on postprandial inflammation (33), despite a well-established relation between fasting hyperglycemia and low-grade inflammation (34). Although we observed a large increase (of 169%) in 6-h IL-6, there was no relation between postprandial IL-6 (4 h and 6 h) and any features of the postprandial glucose or TG response. One possible explanation for this is that postprandial increases in IL-6 may be a consequence of cannulation, which has been previously shown to elicit similar levels of increase in acute inflammation when no meal is consumed (35), rather than the direct effects of the meal.

We observed a modest correlation between fasting GlycA and IL-6, in accordance with previous studies (15, 36), but no correlation between postprandial IL-6 and GlycA, highlighting the divergent mechanisms of postprandial initiation for these 2 inflammatory markers (or the aforementioned impact of cannulation). Indeed, unlike IL-6, GlycA concentrations reflect a composite measure of systemic inflammation (8, 9), to which IL-6, CRP, fibrinogen, and cytokines contribute only negligibly, as to the GlycA signal from NMR (37).

Although the mean postprandial increase in plasma GlycA concentration (4.5%) was small in comparison with IL-6 (169%), when only the participants eliciting an increase in GlycA were examined (60% of the total) the increase was 10.5% (0.14 mmol/L). Given that even small changes in GlycA are associated with risk of disease and mortality (15, 29, 30), these findings highlight a potentially important postprandial inflammatory measure which may have clinical relevance. For example, risk (HR) of future cardiovascular events was 1.31 per 1-SD increment (0.24 mmol/L) in GlycA (after multiple adjustments) (38). In addition, in our trial, participants with the largest postprandial increase in GlycA (>90th percentile; 30% increase from fasting: 1.48 to 1.91 mmol/L), had a 2-fold greater ASCVD risk score (0.034) than the rest of the cohort (0.019; *P* = 0.012); suggesting that individuals that elicit higher postprandial inflammatory responses may be at greater CVD risk.

A strength of the current study is the use of a mixed-nutrient sequential test meal challenge, representing real-life eating patterns. Previous studies have typically focused on single components of the postprandial response—lipemia or glycemia—using supraphysiological and single challenge meals. However, we typically consume multiple mixed-nutrient meals throughout the day which elicit an interrelated lipemic and glycemic response. Indeed, previous studies have shown that the type of fat (39), macronutrient distribution (40, 41), and overall nutrient density (42, 43) of the meal can alter the postprandial inflammatory response.

Owing to the test meal design of our study, we demonstrated, to our knowledge for the first time, that postprandial glycemia and lipemia have an independent and cumulative association with GlycA. This may be mediated by the production of ROS, which may reach higher concentrations during co-occurrence of lipemia and glycemia (44). Our analysis (including multiple regression and ROC curves) suggests that during the co-occurrence of the second glucose peak and peak lipemia, lipemia is a stronger determinant of the inflammatory response than is glycemia. Owing to the 6-h duration of the study, we were unable to determine the kinetics of the lipemic initiation of inflammation; however, the strong correlation between 6-h TG and GlycA is suggestive of a rapid inflammatory response to dietary fat, as previously reported (45).

Although our results demonstrate the key role of lipemia and adiposity in food-induced inflammation, it is important to also target postprandial elevations in glucose in concert with TG, owing to the interacting and overlapping metabolic pathways regulating glucose and TG (46, 47) and the association of fasting and postprandial TG with carbohydrate intake and fasting and postprandial circulating glucose (3, 48). Potential strategies to attenuate fasting and postprandial TG include (49) consumption of low-glycemic foods, high-dose marine omega-3, fiber, and low alcohol intakes alongside lifestyle modifications including exercise in the 12 h preceding meals and consuming larger meals earlier in the day. Strategies to ameliorate the postprandially induced oxidative/inflammatory pathways, including coadministration of polyphenol-/antioxidant-rich foods (50), may also beneficially attenuate postprandial low-grade inflammation.

There is a growing awareness that single measures of postprandial responses typically used (e.g., 0–2 h glucose iAUC and 0–6 h TG iAUC) are an oversimplification and do not reflect different postprandial pathophysiologies. However, postprandial measurements are burdensome for investigators and participants. Therefore, an aim of the current study was to assess the contribution of the different features of the postprandial response in relation to food-induced inflammation to inform future studies and the potential clinical utility of fat-tolerance tests or oral-glucose-tolerance tests to reduce testing burden and to enable the implementation of targeting strategies. Our results demonstrate that the 6-h concentration in GlycA and its 6-h rise from fasting are suitable target measures to determine postprandial inflammation and to discriminate an individual's inflammatory response after sequential meals.

To dissect the key determinants of an individual's postprandial inflammatory response beyond lipemia and glycemia, we applied machine learning, SEM, and MR, exploiting our dense phenotypic data. Participants with greater visceral fat had a bigger (causal) inflammatory response, partly mediated by fasting TG. In accordance with our results, obese individuals have been shown to elicit higher postprandial inflammatory responses (albeit in IL-6) than lean individuals (31).

### Study limitations

The study duration (6) did not allow us to explore the full kinetics of the TG-induced inflammatory response; however, sequential blood sampling for >6 h is challenging for researchers and burdensome for participants. Future studies would benefit from assessing the impact of single compared with sequential test meals, different doses of fat and carbohydrates, as well as different fatty acids and dietary sources to dissect the effect of individual foods, with their inherent nutrient-matrix complexity, on food-induced inflammation. The adjacent GlycA and TG signals on the NMR platform may have affected our observed associations owing to the possibility of overlapping peaks. As previously discussed, the lack of GWAS data on postprandial GlycA, TG, and glucose is a limitation of our MR; however, given that the SEM revealed that fasting GlycA had a strong effect on postprandial GlycA, the results still highlight important relations between postprandial metabolism and postprandial GlycA. Our study also had a limited ethnic diversity, which may be relevant for inflammation (51, 52) and warrants further investigation. In addition, future studies would benefit from measuring the full breadth of inflammatory biomarkers.

### Conclusions

Postprandial inflammation is largely driven by acute elevations in circulating TG. We identified GlycA after mixed meals as a promising candidate biomarker for assessing the food-induced inflammatory response within typical dietary habits. The large interindividual variability in postprandial inflammation, partly mediated by adiposity, highlights the potential for personalized strategies to target obesity and postprandial metabolic responses to reduce low-grade inflammation in preventative health.

## Supplementary Material

nqab132_Supplemental_File

## Data Availability

Data described in the article, code book, and analytic code are held with the Department of Twin Research at King's College London and will be made available using our normal procedures overseen by the Wellcome Trust and its guidelines as part of our core funding. The application is at: https://twinsuk.ac.uk/resources-for-researchers/access-our-data/.

## Notes

Supported by Zoe Global and theWellcome Trust. The Department of Twin Research is supported by Wellcome Trust grant 212904/Z/18/Z, Medical Research Council/British Heart Foundation Ancestry and Biological Informative Markers for Stratification of Hypertension (AIMHY) grant MR/M016560/1, the European Union, the Chronic Disease Research Foundation, Zoe Global, the National Institute for Health Research Clinical Research Facility, and the Biomedical Research Centre (based at Guy's and St Thomas’ National Health Service Foundation Trust in partnership with King's College London). PWF was supported by the European Research Council (ERC-2015-CoG - 681742 NASCENT) and the Swedish Research Council (Distinguished Young Researcher’s Award in Medicine).

The study sponsors (Zoe Global Ltd; JW and GH) contributed as part of the Scientific Advisory Board in the study design and data collection.

MM, TDS, PWF, and SEB contributed equally to this work.

Supplemental Methods, Supplemental Figures 1 and 2, and Supplemental Tables 1–7 are available from the “Supplemental data” link in the online posting of the article and from the same link in the online table of contents at https://academic.oup.com/ajcn/.

Abbreviations used: ASCVD, atherosclerotic cardiovascular disease; CONSORT, Consolidated Standards of Reporting Trials; CRP, C-reactive protein; CVD, cardiovascular disease; DZ, dizygotic; GlycA, glycoprotein acetylation; GWAS, genome-wide association study; iAUC, incremental AUC; IVW, inverse‐variance weighted; MR, Mendelian randomization; MZ, monozygotic; PREDICT, Personalised Responses to Dietary Composition Trial; RF, random forest; ROC, receiver operating characteristic; ROS, reactive oxygen species; rRNA, ribosomal RNA; SEM, structural equation model; SNP, single nucleotide polymorphism; TG, triacylglycerol; T2D, type 2 diabetes; VFM, visceral fat mass.
