# Supplementary material for: Meal-induced inflammation: postprandial insights from the Personalised REsponses to DIetary Composition Trial (PREDICT) study in 1000 participants
Source: Am J Clin Nutr. 2021 Jun 8;114(3):1028–38. doi: 10.1093/ajcn/nqab132 (PMC8408875; doi:10.1093/ajcn/nqab132)
Supplement: nqab132_Supplemental_File [file nqab132_supplemental_file.docx]

**Meal-induced inflammation: Postprandial insights from the PREDICT study in 1,000 participants, Mohsen Mazidi et al.**

**On-line Supplementary Material**

**Supplementary methods**

**Mendelian Randomization**

***Genetic predictors of exposures:*** We retrieved summary data for the association between SNPs and circulating fasting TG from the Global Lipid Genetics Consortium (GLGC) (188,577 adult samples of European ancestry) (1). They included rigorous quality control, imputation to the 1000 Genomes Project panel and adjustments for age and population structure. Persons of European ancestry from 47 studies genotyped with different genome-wide association study arrays (n=94,595) or on the Metabochip array (n=93,982) with imputation to the 1000 Genomes Project reference were studied. In most included studies, blood lipid concentrations had been measured after >8 hours of fasting. Participants on lipid lowering medications were excluded. Traits were adjusted for age, age-squared, sex and principle components, as well as quantile-normalized within each cohort. For genetic association analysis by linear regression, lipid levels were inverse normal-transformed and cohort-wise results combined in fixed effect meta-analysis (1).

For the measures of fasting glucose we used two large GWAS conducted by the MAGIC consortia, which identified multiple genetic loci associated with fasting glucose markers. All participating cohorts in the meta-analyses were of European ancestry. The meta-analyses on fasting glucose from the MAGIC consortium comprised data from 21 different cohorts (2).

If a SNP was unavailable for the outcome GWAS summary statistics, we identified proxy SNPs with a minimum linkage disequilibrium (LD) r2= 0.8. To minimize bias in effect estimates induced by correlation between SNPs, we restricted our genetic instrument to independent SNPs not in linkage disequilibrium (p=0.0001). We refer to a set of SNPs that proxy serum GlycA as “genetic instruments.”

***Genetic predictors of outcomes:*** Genetic associations with fasting GlycA (quantified by nuclear magnetic resonance) were obtained from the largest available extensively genotyped study (among 24,925 adults). More detailed information can be found elsewhere (3).

***Statistics:*** We combined the effect of instruments using inverse variance weighted (IVW) method. Heterogeneity was assessed using Q value for IVW. To address the potential effect of pleiotropic variants on the final effect estimate, we performed sensitivity analysis including weighted median (WM) and MR-Egger. Sensitivity analysis was conducted using the leave-one-out method to identify instruments that might drive the MR results (4). The WM estimate provides correct estimates as long as SNPs accounting for ≥50% of the weight are valid instruments. Inverse variance is used to weight the variants and bootstrapping is applied to estimate the CIs (4). MR-Egger is able to make estimates even under the assumption that all SNPs are invalid instruments, as long as the assumption of instrument strength independent of direct effect (InSIDE) is satisfied (4). However, the InSIDE assumption cannot be easily verified. Average directional pleiotropy across genetic variants was assessed from the *p* value of the intercept term from MR-Egger. Causal estimates in MR-Egger are less precise than those obtained by using IVW MR. Analysis using MR-Egger has a lower false-positive rate, but a higher false-negative rate, than IVW i.e. it has a lower statistical power(5).

Heterogeneity between individual genetic variant estimates was assessed by the use of the Q′ heterogeneity statistic (6). The Q′ statistic uses modified 2^nd^ order weights that are a derivation of a Taylor series expansion, taking into account the uncertainty in both numerator and denominator of the instrumental variable ratio (6).

**Sensitivity analysis:** As sensitivity analysis, we used MR-Egger and MR pleiotropy residual sum and outlier (MR-PRESSO) test (5). MR-Egger and MR-PRESSO may provide correct estimates as long as the instrument strength independent of direct effect assumption is satisfied. MR-Egger can be imprecise, particularly if the associations for SNPs on exposure are similar, or the number of genetic instruments is low (5). A non-null MR-Egger intercept suggests that the IVW estimate is invalid. MR-Egger does not explicitly identify outliers. MR-PRESSO detects, and if necessary, corrects for potentially pleiotropic outliers (5). The MR-PRESSO framework detects effect estimates that are outliers and removes them from the analysis by regressing the variant-outcome associations on variant-exposure associations. A global heterogeneity test is then implemented to compare the observed distance between residual sums of squares of all variants to the regression line with the distance expected under the null hypothesis of no pleiotropy (6). Furthermore, MR-Robust Adjusted Profile Score (RAPS) was applied. This method can correct for pleiotropy using robust adjusted profile scores. We consider as results, causal estimates that agreed in direction and magnitude across MR methods, passed nominal significance in IVW MR, and did not show evidence of bias from horizontal pleiotropy (variant has an effect on other traits outside of the pathway of the GlycA) using heterogeneity tests.

**Structural equation modeling**

Structural equation modelling (SEM) was used to test the overall model fit and relationships between sets of variables which were selected from machine learning to understand the underlying cause of the postprandial GlycA (6h) response. SEMs are able to test the fit of the defined model based on the observed covariance between the variables. We fitted our model under a maximum likelihood framework using covariance matrices. Relative model fit was assessed using the comparative fit index (CFI) and the Tucker–Lewis index (TLI), with values ranging from 0 (no fit) to 1 (perfect fit); a model with a ‘good’ fit typically requires both indices to exceed 0.95 (7). Absolute fit was assessed using root mean square error of approximation (RMSEA). This ranges from 0 to 1, with 0 indicating a perfect fit (7, 8). A poorly fitting model is typically defined by RMSEA >0.06 (7). CFI, TLI and RMSEA were not used to formally determine adequacy of fit, as their use in this context is controversial and there is limited consensus on appropriate cut-off values because each index is affected differently by degrees of freedom, model complexity and sample size; it is, however, standard practice to report these along with the χ2.

In this study assumptions of the Pearson correlations, regression analysis, repeated measures ANOVA, random forest models and structural equation modeling have been met. The natural logarithm of IL-6 for all three time points (+1) was calculated to normalize data distributions.

**References:**

1. Willer CJ, Schmidt EM, Sengupta S, Peloso GM, Gustafsson S, Kanoni S, Ganna A, Chen J, Buchkovich ML, Mora S, et al. Discovery and refinement of loci associated with lipid levels. Nature genetics 2013;45(11):1274-83. doi: 10.1038/ng.2797.

2. Dupuis J, Langenberg C, Prokopenko I, Saxena R, Soranzo N, Jackson AU, Wheeler E, Glazer NL, Bouatia-Naji N, Gloyn AL, et al. New genetic loci implicated in fasting glucose homeostasis and their impact on type 2 diabetes risk. Nature genetics 2010;42(2):105-16. doi: 10.1038/ng.520.

3. Shin SY, Fauman EB, Petersen AK, Krumsiek J, Santos R, Huang J, Arnold M, Erte I, Forgetta V, Yang TP, et al. An atlas of genetic influences on human blood metabolites. Nature genetics 2014;46(6):543-50. doi: 10.1038/ng.2982.

4. Bowden J, Davey Smith G, Haycock PC, Burgess S. Consistent Estimation in Mendelian Randomization with Some Invalid Instruments Using a Weighted Median Estimator. Genetic epidemiology 2016;40(4):304-14. doi: 10.1002/gepi.21965.

5. Bowden J, Del Greco MF, Minelli C, Davey Smith G, Sheehan N, Thompson J. A framework for the investigation of pleiotropy in two-sample summary data Mendelian randomization. Statistics in medicine 2017;36(11):1783-802. doi: 10.1002/sim.7221.

6. Verbanck M, Chen CY, Neale B, Do R. Detection of widespread horizontal pleiotropy in causal relationships inferred from Mendelian randomization between complex traits and diseases. Nature genetics 2018;50(5):693-8. doi: 10.1038/s41588-018-0099-7.

7. Shi D, Lee T, Maydeu-Olivares A. Understanding the Model Size Effect on SEM Fit Indices. Educational and psychological measurement 2019;79(2):310-34. doi: 10.1177/0013164418783530.

8. Xia Y, Yang Y. RMSEA, CFI, and TLI in structural equation modeling with ordered categorical data: The story they tell depends on the estimation methods. Behavior research methods 2019;51(1):409-28. doi: 10.3758/s13428-018-1055-2.

**Supplementary Figure 1.** Consort Diagram


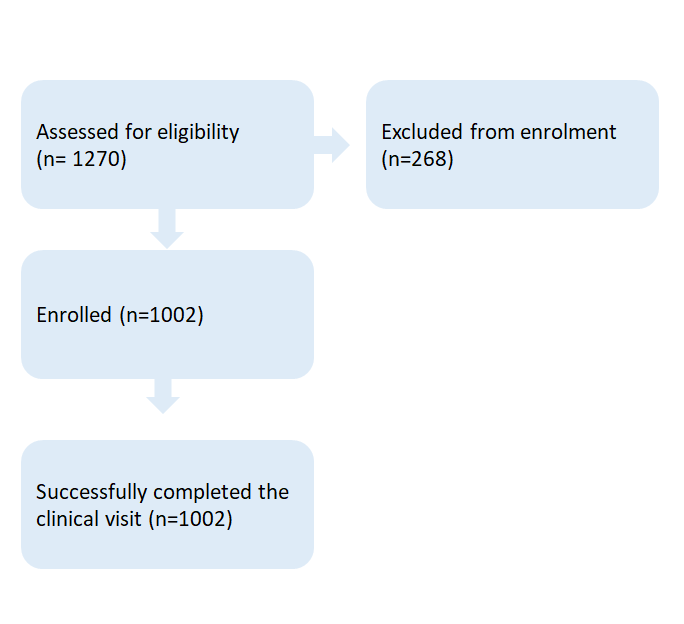


| **Supplementary Table 1: Postprandial changes in IL-6 and GlycA stratified by sex (n=1002)** | | | | | |
| --- | --- | --- | --- | --- | --- |
|  | | **Fasting** | **4h** | **6h** | **Interaction**  **p-value** |
| **IL-6** | **Male** | 0.66±0.67 | 1.97±2.40 | 2.25±3.26 | <0.001 |
|  | **Female** | 0.74±1.30 | 2.49±2.52 | 3.38±3.94 |  |
| **GlycA** | **Male** | 1.36±0.18 | 1.44±0.24 | 1.48±0.30 | <0.001 |
|  | **Female** | 1.31±0.17 | 1.36±0.22 | 1.34±0.26 |  |
| GlycA : Glycoprotein Acetyls, IL-6: Interleukin 6 | | | | | |

| **Supplementary Table 2: Postprandial changes in IL-6 and GlycA stratified by age (n=1002)** | | | | | |
| --- | --- | --- | --- | --- | --- |
|  | | **Fasting** | **4h** | **6h** | **Interaction**  **p-value** |
| **IL-6** | **18-30 (years)** | 0.66±1.19 | 1.76±1.70 | 3.12±3.54 | 0.086 |
|  | **30-40**  **(years)** | 0.64±0.96 | 2.08±1.92 | 2.96±3.06 |  |
|  | **40-50**  **(years)** | 0.61±0.46 | 2.54±2.96 | 3.58±3.26 |  |
|  | **50-65**  **(years)** | 0.85±1.47 | 2.49±2.50 | 3.90±4.32 |  |
| **GlycA** | **18-30**  **(years)** | 1.30±0.17 | 1.33±0.19 | 1.31±0.20 | <0.001 |
|  | **30-40**  **(years)** | 1.30±0.17 | 1.34±0.21 | 1.33±0.27 |  |
|  | **40-50**  **(years)** | 1.31±0.19 | 1.37±0.24 | 1.36±0.28 |  |
|  | **50-65**  **(years)** | 1.35±0.17 | 1.42±0.23 | 1.44±0.29 |  |
| GlycA: Glycoprotein Acetyls, IL-6: Interleukin 6 | | | | | |

| **Supplementary Table 3: Correlation between fasting and postprandial (4 and 6h) IL-6 and GlycA concentrations. (n=1002)** | | | | | | | |
| --- | --- | --- | --- | --- | --- | --- | --- |
| **Variables** | | **Fasting IL-6** | **Postprandial 4h IL-6** | **Postprandial 4h IL-6** | **Fasting GlycA** | **Postprandial 4h GlycA** | **Postprandial 6h GlycA** |
| **Fasting IL-6** | coefficient | 1 | 0.303** | 0.237** | 0.349** | 0.309** | 0.275** |
|  | P-value |  | <0.001 | <0.001 | <0.001 | <0.001 | <0.001 |
| **Postprandial 4h IL-6** | coefficient | 0.303** | 1 | 0.575** | 0.064* | 0.061 | 0.046 |
|  | P-value | <0.001 |  | <0.001 | 0.047 | 0.056 | 0.155 |
| **Postprandial 4h IL-6** | coefficient | 0.237** | 0.575** | 1 | 0.032 | 0.028 | -0.019 |
|  | P-value | <0.001 | <0.001 |  | 0.327 | 0.397 | 0.552 |
| **Fasting GlycA** | coefficient | 0.349** | 0.064* | 0.032 | 1 | 0.873** | 0.803** |
|  | P-value | <0.001 | 0.047 | 0.327 |  | <0.001 | <0.001 |
| **Postprandial 4h GlycA** | coefficient | 0.309** | 0.061 | 0.028 | 0.873** | 1 | 0.905** |
|  | P-value | <0.001 | 0.056 | 0.397 | <0.001 |  | <0.001 |
| **Postprandial 6h GlycA** | coefficient | 0.275** | 0.046 | -0.019 | 0.803** | 0.905** | 1 |
|  | P-value | <0.001 | 0.155 | 0.552 | <0.001 | <0.001 |  |
| GlycA : Glycoprotein Acetyls, IL-6: interleukin 6 | | | | | | | |

| **Supplementary Table 4: Area Under Curve, Lower Bound and Upper Bound of the associations between the first and second postprandial glucose peak and postprandial TG peak with postprandial inflammation (n=1,002)** | | | | |
| --- | --- | --- | --- | --- |
| **Variables** |  | **Area Under Curve** | **Lower Bound** | **Upper Bound** |
| **Postprandial Inflammation** | **Triglyceride Peak** | 0.884 | 0.860 | 0.908 |
|  | **First Glucose Peak** | 0.632 | 0.593 | 0.671 |
|  | **Triglyceride Peak and First Glucose Peak** | 0.885 | 0.862 | 0.909 |
| **Postprandial Inflammation** | **First Glucose Peak** | 0.632 | 0.593 | 0.671 |
|  | **Second Glucose Peak** | 0.57 | 0.529 | 0.610 |
|  | **First Glucose Peak and Second Glucose Peak** | 0.632 | 0.593 | 0.671 |
| **Postprandial Inflammation** | **Triglyceride Peak** | 0.880 | 0.856 | 0.904 |
|  | **Second Glucose Peak** | 0.585 | 0.546 | 0.624 |
|  | **Triglyceride Peak and Second Glucose Peak** | 0.881 | 0.857 | 0.905 |

| **Supplementary Table 5: Baseline Dietary factors (n=1002)** | | |
| --- | --- | --- |
| **Variables** | **Median** | **(25^th^-75^th^)** |
| **Alpha carotene(mg)** | 536.00 | 187.00 - 558.00 |
| **Alcohol(g)** | 6.00 | 2.00 – 13.00 |
| **Beta carotene(mg)** | 3022.50 | 2155.25 – 4117.25 |
| **Calcium(mg)** | 676.00 | 526.00 – 836.50 |
| **Carotene - total(mg)** | 3403.00 | 2420.50 – 4632.50 |
| **Carbohydrate - total(g)** | 181.00 | 143.00 – 228.00 |
| **Cholesterol(mg)** | 235.50 | 173.25 – 308.00 |
| **Chloride(mg)** | 3470.50 | 2677.25 – 4339.00 |
| **Copper(g)** | 1.00 | 1.00 – 1.00 |
| **Non Starch Polysaccharides (g)** | 16.00 | 13.00 – 20.00 |
| **Iron(mg)** | 11.00 | 9.00 – 14.00 |
| **Total folate(mg)** | 263.00 | 205.00 – 326.00 |
| **Carbohydrate - fructose(g)** | 18.00 | 13.00 – 24.00 |
| **Carbohydrate - galactose(g)** | 0.00 | 0.00 – 1.00 |
| **Carbohydrate - glucose(g)** | 17.00 | 12.00 – 22.00 |
| **Iodine(mg)** | 105.50 | 78.25 – 136.00 |
| **Potassium(mg)** | 3121.00 | 2582.75 – 3767.75 |
| **Energy(kcal)** | 1648.50 | 1313.75 – 1995.50 |
| **Carbohydrate - lactose(g)** | 8.00 | 3.00 – 11.00 |
| **Carbohydrate - maltose(g)** | 2.00 | 1.00 – 3.00 |
| **Magnesium(mg)** | 307.50 | 245.00 – 374.00 |
| **Manganese(mg)** | 3.00 | 2.00 – 4.00 |
| **Sodium(mg)** | 2329.00 | 1799.25 – 2981.75 |
| **Niacin(mg)** | 21.00 | 17.00 – 26.00 |
| **Phosphorus(mg)** | 1230.00 | 992.75 – 1458.25 |
| **Protein(mg)** | 74.00 | 58.00 – 89.00 |
| **Vitamin A - retinol(mg)** | 255.50 | 166.25 – 405.25 |
| **Vitamin A - retinol equivalents(mg)** | 864.00 | 648.25 – 1238.75 |
| **Vitamin B2(mg)** | 2.00 | 1.00 – 2.00 |
| **Selenium(mg)** | 61.00 | 47.00 – 78.00 |
| **Carbohydrate - starch(g)** | 95.00 | 70.00 – 122.00 |
| **Carbohydrate - sucrose(g)** | 35.00 | 25.00 – 44.00 |
| **Vitamin B1(mg)** | 1.00 | 1.00 – 2.00 |
| **Nitrogen(mg)** | 12.00 | 9.00 – 14.00 |
| **Carbohydrate - sugars (total)(g)** | 84.00 | 61.25 – 102.00 |
| **Vitamin B12(g)** | 5.00 | 4.00 – 8.00 |
| **Vitamin B6(g)** | 2.00 | 2.00 – 2.00 |
| **Vitamin C(mg)** | 105.00 | 77.00 – 136.00 |
| **Vitamin D(mg)** | 2.00 | 2.00 – 4.00 |
| **Vitamin E - alpha tocopherol equivalents(mg)** | 10.00 | 8.00 – 12.00 |
| **Zinc(mg)** | 8.00 | 7.00 – 10.00 |
| **Fat - total(g)** | 66.00 | 50.00 – 83.00 |
| **Monounsaturated fatty acids(g)** | 24.00 | 18.00 – 30.00 |
| **Polyunsaturated fatty acids(g)** | 13.00 | 10.00 – 17.00 |
| **Saturated fatty acids(g)** | 23.00 | 17.00 – 29.75 |

| **Supplementary Table 6: Individual edge effect estimates for the underlying mechanism of postprandial GlycA (6h) (n=1002)** | | | | | |
| --- | --- | --- | --- | --- | --- |
| **Variables in the structural equation models** | | | **Standardized beta** | **Standard**  **error** | **P-value** |
| Visceral fat |  | Total cholesterol to HDL ratio | 0.466 | 0.028 | <0.001 |
| Visceral fat | -----> | Fasting triglyceride | 0.183 | 0.028 | <0.001 |
| Total cholesterol to high-density lipoprotein ratio | -----> | Fasting triglyceride | 0.499 | 0.028 | <0.001 |
| Visceral fat | -----> | Total Bilirubin | -0.094 | 0.031 | 0.003 |
| Fasting glucose | -----> | Fasting GlycA | 0.028 | 0.019 | 0.135 |
| Microbiome (first PCA) | -----> | Fasting GlycA | 0.012 | 0.019 | 0.544 |
| Fasting triglyceride | -----> | Fasting GlycA | 0.572 | 0.024 | <0.001 |
| Visceral fat | -----> | Fasting GlycA | 0.173 | 0.022 | <0.001 |
| Total cholesterol to high-density lipoprotein ratio | -----> | Fasting GlycA | 0.161 | 0.025 | <0.001 |
| Total Bilirubin | -----> | Fasting GlycA | -0.103 | 0.019 | <0.001 |
| Total Bilirubin | -----> | GlycA (6h) | -0.006 | 0.021 | 0.772 |
| Total cholesterol to high-density lipoprotein ratio | -----> | GlycA (6h) | 0.111 | 0.027 | <0.001 |
| Visceral fat | -----> | GlycA (6h) | 0.119 | 0.024 | <0.001 |
| Fasting triglyceride | -----> | GlycA (6h) | 0.236 | 0.032 | <0.001 |
| Microbiome (first PCA) | -----> | GlycA (6h) | 0.030 | 0.020 | 0.143 |
| Fasting glucose | -----> | GlycA (6h) | 0.016 | 0.020 | 0.445 |
| Fasting GlycA | -----> | GlycA (6h) | 0.415 | 0.034 | <0.001 |
| GlycA : Glycoprotein Acetyls | | | | | |

| **Supplementary Table 7: Results of the Mendelian Randomization (MR) analysis for the impact of body fat, fasting triglyceride and fasting glucose on fasting GlycA** | | | | | | | | | | |
| --- | --- | --- | --- | --- | --- | --- | --- | --- | --- | --- |
| **Exposures** | **MR** | | | | **Heterogeneity** | | | **Pleiotropy** | | |
|  | **Method** | **beta** | **SE** | **p** | **Method** | **Q** | **P-value** | **Intercept** | **SE** | **p** |
| **Body fat** | **MR Egger** | 0.318 | 0.116 | 6.930e-03 | **MR-Egger** | 296.85 | 0.108 | -0.0021 | 0.0023 | 0.364 |
|  | **WM** | 0.235 | 0.055 | 2.203e-05 |  |  |  |  |  |  |
|  | **IVW** | 0.217 | 0.037 | 6.634e-09 | **IVW** | 297.76 | 0.109 |  |  |  |
|  | **RAPS** | 0.234 | 0.037 | 6.221e-10 |  |  |  |  |  |  |
| **Fasting triglyceride** | **MR Egger** | 0.553 | 0.076 | 2.421e-09 | **MR-Egger** | 102.88 | 3.358e-05 | -0.0035 | 0.003 | 0.335 |
|  | **WM** | 0.463 | 0.060 | 1.245e-14 |  |  |  |  |  |  |
|  | **IVW** | 0.494 | 0.046 | 6.915e-26 | **IVW** | 104.75 | 2.958e-05 |  |  |  |
|  | **RAPS** | 0.494 | 0.043 | 1.613e-30 |  |  |  |  |  |  |
| **Fasting glucose** | **MR Egger** | 0.0001 | 0.239 | 0.999 | **MR-Egger** | 103.07 | 2.129e-09 | -0.0046 | 0.0070 | 0.515 |
|  | **WM** | -0.008 | 0.101 | 0.931 |  |  |  |  |  |  |
|  | **IVW** | -0.132 | 0.126 | 0.294 | **IVW** | 104.46 | 2.382e-09 |  |  |  |
|  | **RAPS** | -0.055 | 0.093 | 0.555 |  |  |  |  |  |  |
| WM: Weighted median, IVW: Inverse variance weighted, SE: standard error, beta: beta-coefficients. RAPS: MR-Robust Adjusted Profile Score, this method can correct for pleiotropy using robust adjusted profile scores. | | | | | | | | | | |


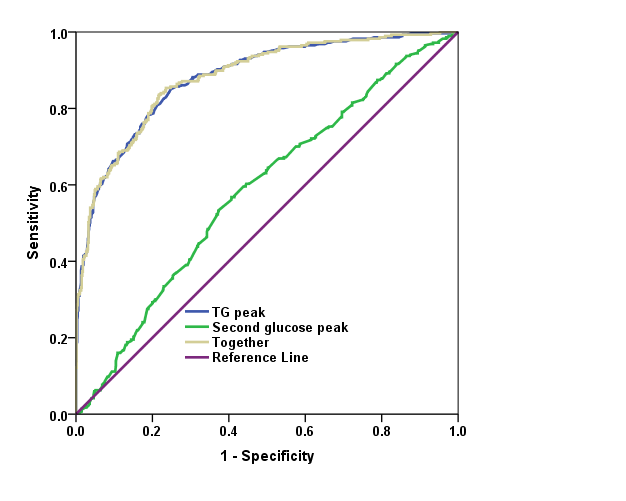
**
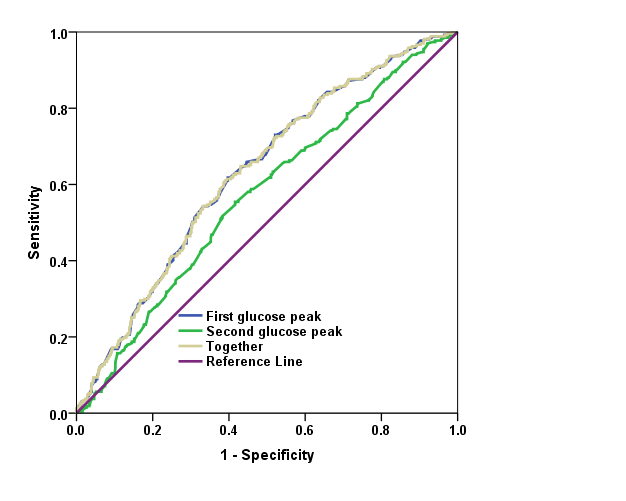
**
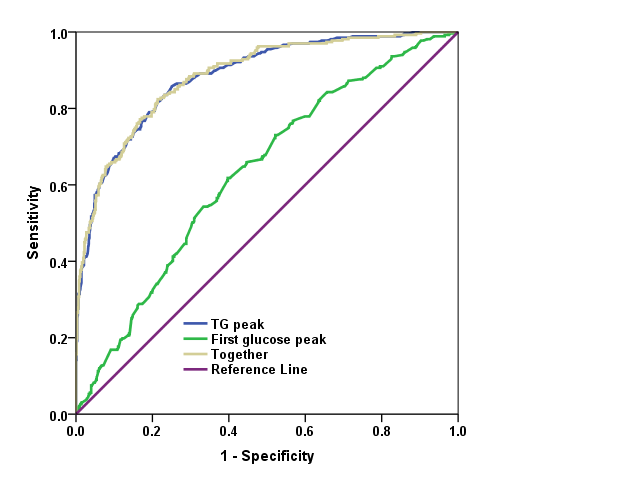


**Supplementary Figure 2**: Associations between the first and second postprandial glucose peak and postprandial TG peak with postprandial inflammation. A) Receiver operator characteristic curves illustrating the predictive utility of postprandial TG peak (TG_peak6h_ ) and first postprandial glucose peak (Glu_peak2h_) to discriminate the bottom 70% from the top 30% of the cohort based on 6h GlycA, B) Receiver operator characteristic curves illustrating the predictive utility of the first and second postprandial glucose peak to discriminate the bottom 70% from the top 30% of the cohort based on 6h GlycA, C) Receiver operator characteristic curves illustrating the predictive utility of the second postprandial glucose peak and postprandial TG peak (TG_peak6h_).
